# Operating a patient medicines helpline: a survey study exploring current practice in England using the RE-AIM evaluation framework

**DOI:** 10.1186/s12913-018-3690-9

**Published:** 2018-11-20

**Authors:** Matt Williams, Abbie Jordan, Jenny Scott, Matthew D. Jones

**Affiliations:** 1 Department of Pharmacy & Pharmacology, University of Bath, 5 West, Claverton Down, Bath, BA2 7AY UK; 2 Department of Psychology, University of Bath, 10 West, Claverton Down, Bath, BA2 7AY UK

**Keywords:** Patient medicines helplines, RE-AIM, National Health Service, Medicines information, Drug information, Hospital pharmacy, Clinical audit

## Abstract

**Background:**

Patient medicines helplines provide a means of accessing medicines-related support following hospital discharge. However, it is unknown how many National Health Service (NHS) Trusts currently provide a helpline, nor how they are operated. Using the RE-AIM evaluation framework (*Reach, Effectiveness, Adoption, Implementation, and Maintenance*), we sought to obtain key data concerning the provision and use of patient medicines helplines in NHS Trusts in England. This included the extent to which the delivery of helplines meet with national standards that are endorsed by the Royal Pharmaceutical Society (standards pertaining to helpline access, availability, and promotion).

**Methods:**

An online survey was sent to Medicines Information Pharmacists and Chief Pharmacists at all 226 acute, mental health, specialist, and community NHS Trusts in England in 2017.

**Results:**

*Adoption*: Fifty-two percent of Trusts reported providing a patient medicines helpline (acute: 67%; specialist: 41%; mental health: 29%; community: 18%). *Reach*: Helplines were predominantly available for discharged inpatients, outpatients, and carers (98%, 95% and 93% of Trusts, respectively), and to a lesser extent, the local public (22% of Trusts). The median number of enquiries received per week was five. *Implementation*: For helpline access, 54% of Trusts reported complying with all ‘satisfactory’ standards, and 26% reported complying with all ‘commendable’ standards. For helpline availability, the percentages were 86% and 5%, respectively. For helpline promotion, these percentages were 3% and 40%. One Trust reported complying with all standards. *Maintenance*: The median number of years that helplines had been operating was six. *Effectiveness*: main perceived benefits included patients avoiding harm, and improving patients’ medication adherence.

**Conclusions:**

Patient medicines helplines are provided by just over half of NHS Trusts in England. However, the proportion of mental health and community Trusts that operate a helpline is less than half of that of the acute Trusts, and there are regional variations in helpline provision. Adherence to the national standards could generally be improved, although the lowest adherence was regarding helpline promotion. Recommendations to increase the use of helplines include increasing the number of promotional methods used, the number of ways to contact the service, and the number of hours that the service is available.

**Electronic supplementary material:**

The online version of this article (10.1186/s12913-018-3690-9) contains supplementary material, which is available to authorized users.

## Background

Patients often experience changes to their medicines regimen while they are in hospital, and it is healthcare policy in the United Kingdom (UK) to ensure that patients’ medicines are managed optimally after discharge from secondary care [1, 2]. However, UK and international research suggest that a substantial proportion of patients who have been discharged from hospital subsequently experience medicines-related problems [3–7]. For example, Lee et al. [3] conducted a study which involved interviewing ninety-six patients after being discharged from one of six acute hospitals in the North-West of England. They found that 36% of patients experienced problems with their medication following discharge, particularly around side effects (63%), and that 26% had actually sought or been given help following discharge, mainly from their general practitioner. Relatedly, UK and international research also show that patients often lack knowledge of their medications following discharge from hospital, particularly around side effects [8–13], and that many patients report not receiving important medicines-related information [14–16]. Results from the 2017 UK National Health Service (NHS) Adult Inpatient Survey found that 30% of 46,795 patients reported that they were not provided with completely clear written or printed information about their medicines, and 43% of 43,719 patients did not recall receiving any information from staff about side effects to look out for when they returned home [17]. Another evident problem which patients may also experience following hospital discharge are medicines-related errors, such as prescribing errors and incorrect or missing information on discharge summary documents [18–20]. In sum, discharge from hospital presents a potentially confusing and/or risky time for the many patients who have recently experienced changes to their medicines.

In many countries, medicines information (MI) services have been established to support patients and the public with questions about their medication [21–25]. In the UK, patient medicines helplines have become available from a number of hospital pharmacies to provide medicines-related support for patients who have received care within secondary healthcare [26, 27]. Patient medicines helplines are typically operated by pharmacy professionals (pharmacists and pharmacy technicians registered with the General Pharmaceutical Council) who specialise in the provision of MI services (from here on, referred to as MI pharmacy professionals) [28]. The first patient medicines helpline in the UK was established in 1992, with the aim of improving patients’ knowledge and use of their medicines [29]. In 2007, the Healthcare Commission in the UK reported that, of the 173 acute and specialist NHS Trusts^1^ in England, 64% operated a patient medicines helpline, and of 42 mental health Trusts in England and Wales, 31% operated this service [27, 30]. However, over ten years later, it is unknown how many NHS Trusts currently provide a patient medicines helpline.

Recently, several service evaluation studies have been published that provide descriptive information about patient medicines helplines, typically reporting the types of calls received and user satisfaction ratings. Such studies suggest that enquiries predominantly concern issues such as adverse effects, administration and dosage, and interactions [26, 28, 31–33]. Enquiries can also result in patients avoiding harm, such as by highlighting medicines-related errors so that they can be corrected [26, 31, 34]. Evaluations of patients’ and carers’ experiences of using medicines helplines using self-report surveys suggest that services are thorough and that they found the advice useful, that they felt confident with the information they received, and felt reassured as a result [32–34]. Consequently, patient medicines helplines offer a means of providing medicines-related support following discharge from secondary care, which users find satisfactory [34]. Moving beyond the individual, an additional proposed benefit of patient medicines helplines is that they may reduce the burden on primary care and emergency services [35]. Evaluation studies suggest that, if patient medicines helplines did not exist, enquirers would typically contact their general practitioner to resolve their medicines-related queries [34]. Providing access to a medicines helpline accords with healthcare policy regarding the importance of patients having access to information about their care, and being involved in care-related decisions [36–39]. Additionally, a priority of UK healthcare policy is to improve patients’ transitions of care so they are able to manage their own health, and know how to access healthcare support [37–40].

Although service evaluation studies have been conducted to examine patient medicines helplines, to date, no healthcare evaluation frameworks have been applied for the evaluation of this service. Evaluation frameworks are considered to be beneficial, since they provide a structured and guided approach to evaluating an overall program or intervention, and are typically evidence-based [41]. A widely used framework is RE-AIM, which was first published in 1999 [42, 43] and is recommended in Medical Research Council guidance [44]. Whereas most studies focus upon the effectiveness of an intervention, RE-AIM comprises five dimensions that are considered important for evaluating the public health impact of interventions. These are:

- Reach (proportion and representativeness of the population receiving the intervention);
- Effectiveness (assessment of the positive and negative consequences of an intervention);
- Adoption (proportion and representativeness of settings that adopt an intervention);
- Implementation (extent to which an intervention is delivered as intended);
- Maintenance (extent to which an intervention becomes a relatively stable, enduring part of the behavioural repertoire of an individual/organisation).

The ‘RE’ dimensions are primarily concerned with the impact on the individual (e.g., whether an intervention is beneficial for the people receiving it, and how many individuals who could benefit from it are receiving it). The ‘AIM’ dimensions are primarily concerned with the impact of an intervention at the level of the intervention setting (e.g., whether sites that could offer the intervention, offer it; whether the intervention is being offered as intended in sites that offer the intervention, and whether this is stable over time). Glasgow and colleagues argued that whilst the ‘AIM’ dimensions are less often studied, they are equally important factors in determining the impact of an intervention [45].

The main aim of this study was to obtain key data concerning the provision and usage of patient medicines helplines in NHS Trusts in England. The RE-AIM framework was considered particularly useful to achieve this, since patient medicines helpline services could potentially be adopted by all NHS Trusts with the aim of being available for all of a Trust’s patients (*Adoption* and *Reach*). Additionally, national standards for setting up and operating a patient medicines helpline in the UK have recently been developed, and are endorsed by the Royal Pharmaceutical Society [46]. This provides the opportunity to evaluate the extent that current practice in the provision of patient medicines helplines meets these standards (*Implementation*).

Using the RE-AIM framework, the following five study objectives were developed (re-ordered so that ‘AIM’ precedes ‘RE’, since the existence and delivery of an intervention precedes its use and perceived effectiveness):

1. Establish the percentage of NHS Trusts in England that provide a patient medicines helpline, including the percentages by region, and explore the reasons why some Trusts do not provide this service (*Adoption*).
2. Examine how patient medicines helplines are operated in England, by comparing how current practice meets with national standards for operating patient medicines helplines (*Implementation*).
3. Establish the average number of years that Trusts have operated patient medicines helplines, and the reasons why some Trusts stopped operating a helpline (*Maintenance*).
4. Establish for whom patient medicines helpline services are available, and the average number of enquiries received per week (*Reach*).
5. Establish pharmacy professionals’ perceptions as to the benefits that their patient medicines helpline can have (a proxy measure for *Effectiveness*).

## Method

### Design

This study involved the use of cross-sectional surveys to establish the provision, usage, and current practice in the operation of patient medicines helplines in NHS Trusts in England.

### Participants

Inclusion criteria required participants to be either an MI pharmacy professional at an acute, mental health, specialist or community NHS Trust within England whose role involved operating a patient medicines helpline service at their NHS Trust, or a Chief Pharmacist at an acute, mental health, specialist or community NHS Trust within England that operates a patient medicines helpline service. These two professional groups were chosen because MI pharmacy professionals see first-hand the benefits of medicines helplines for patients, and Chief Pharmacists may be better placed to provide a perspective as to how medicines helplines are beneficial within the wider organisation. Additionally, both groups were considered to have insight regarding the operation of their patient medicines helpline service.

At the time of data collection (February–May 2017) 226 NHS Trusts were eligible to be included in the survey. Regional Medicines Information (MI) centres were not invited to participate, since they were contacted prior to data collection and none provided a regional patient medicines helpline that is separate from an NHS Trust.

### Materials and procedure

#### Developing the data collection tools

Two online surveys were developed using SurveyMonkey [47]. SurveyMonkey is a platform for creating online surveys that is compliant with UK data protection laws, and has been used in other pharmacy practice survey research [48, 49]. Best practice guidance for developing and conducting online surveys was sought and adopted during the design and data collection phases of this study [50, 51]. This included writing survey questions and answer options, and considering ethical issues such as providing participants with information about the study and obtaining consent.

Survey 1 was developed to be completed by a lead MI pharmacy professional at each NHS Trusts (or delegated deputy). This was because Survey 1 was tailored to ask questions about the actual operation of the helpline (e.g., the average number of calls per week, and what the advertised hours are), and MI pharmacy professionals typically perform this role. However, if no-one from the MI team decided to participate, or if the Trust did not have an MI team, Survey 1 was instead sent to the Trust’s Chief Pharmacist to complete.

Firstly, Survey 1 sought to establish whether each NHS Trust provides a patient medicines helpline service (*RE-AIM Adoption*). For those Trusts that did not provide a helpline, subsequent questions within Survey 1 focussed on exploring this in more detail (e.g., whether they ever provided a helpline, and if so, the reason/s why the helpline stopped; and the reason/s why their Trust does not currently provide a helpline service). For NHS Trusts that did provide a helpline service, subsequent questions within Survey 1 explored the operation and usage of the service, structured by the remaining RE-AIM dimensions.

To measure *RE-AIM Implementation*, the sections of the national standards for operating patient medicines helplines [46] pertaining to access, availability, and promotion of patient medicines helplines were developed in to questions for inclusion in the survey. The standards for helpline access, availability, and promotion were used, since these sections are most likely to impact helpline service users (other sections pertain to use of standard operating procedures, use of information and professional support, and quality and risk). The standards are separated in to ‘satisfactory’ and ‘commendable’ aspects of helpline operation, and both types were included in the survey.

For *RE-AIM Maintenance*, participants were asked to report the year that their helpline service was set up, so that this information could be used to establish the average length of time that helplines have been running.

For *RE-AIM Reach*, participants were asked to report who could use the helpline service, and the number of enquiries received to the helpline service per week.

For *RE-AIM Effectiveness*, MI Pharmacy professionals’ and Chief Pharmacists’ perceptions as to the benefits of patient medicines helpline services were sought. A list of potential benefits of patient medicines helplines has been developed by the same small working group of proponents of the service that developed the national standards, and have also been endorsed by the Royal Pharmaceutical Society [35]. These proposed benefits were included in Survey 1, and were the primary feature of Survey 2. Participants’ options were to rate each item as having ‘major benefit’ or ‘minor benefit’/‘no benefit’. Participants were also given the option to report any additional perceived benefits that were not included in the list.

Survey 2 was developed to be completed by Chief Pharmacists (or delegated deputy) at Trusts that operate a medicines helpline, where Survey 1 had already been completed by an MI pharmacy professional. The aim of Survey 2 was to explore Chief Pharmacists’ perspectives as to how patient medicines helplines are beneficial, since Chief Pharmacists may be more likely to take a wider organisational view than those involved in the day-to-day operation of the helpline service. The primary feature of Survey 2 was therefore the *RE-AIM Effectiveness* section of Survey 1.

Overall, survey questions primarily consisted of either yes/no or multiple-choice answers, although some questions also provided free-text boxes. The questions and response options for Survey 1 and Survey 2 are provided in Additional file 1.

#### Pre-test and pilot

Following recommended methods [52], a pre-test of the survey was conducted, with three pharmacists with expertise in the area of patient medicines helplines. The aim of the pre-test was to assess the content, length and format, and to identify problems that may interfere with respondents completing the survey consistently and accurately. Amendments were made based upon the feedback of the pre-test.

Additionally, prior to study commencement, a pilot study was conducted. The pilot involved collecting survey data using a randomly selected 10% of the main study sample, ensuring that Trust type and geographical coverage of England were represented. The results of the pilot suggested that no changes were necessary, so data from the pilot were included in the final results.

#### Data collection

**Fig. 1:**
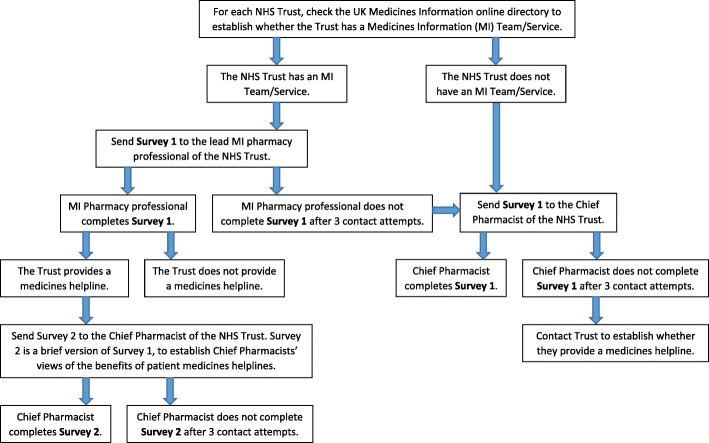
Data collection procedure

Figure 1 shows the procedure for collecting data using the two surveys.

Data were collected between February–May 2017. Survey 1 was sent to MI pharmacy professionals at all acute, mental health, specialist, and community NHS Trusts in England, via email. If Survey 1 was not completed by an MI pharmacy professional, it was sent to the Chief Pharmacist of the NHS Trust, via email. If Survey 1 was completed by an MI pharmacy professional, and if the Trust reported providing a patient medicines helpline, the Chief Pharmacist of the Trust received Survey 2. For all participants, three reminder emails were sent if there was no response, within two weekly intervals. Non-responders were contacted to establish whether or not their Trust provided a helpline. Participants were informed that by completing the survey, they would have the option of being included in a prize draw to win a £25 gift voucher.

#### Data analysis

Data were analysed using SPSS version 23, to primarily produce descriptive statistics (e.g., percentages of NHS Trusts complying with the standards). Chi square tests of independence were used to examine the relationships between Chief Pharmacists’ and MI pharmacy professionals’ ratings of the benefits of patient medicines helplines. To establish the percentage of NHS Trusts which provide a patient medicines helpline by region of England, an official list of NHS Trusts within ten regions of England was used [53].

## Results

### Response rates

Out of 226 NHS Trusts, 202 completed Survey 1 (89%). Of these, 127 (63%) were completed by an MI pharmacy professional, and sixty-two (31%) were completed by a Chief Pharmacist (thirteen did not disclose their job title; 6%). The remaining 11% of Trusts were contacted to establish whether they operated a patient medicines helpline, with all such trusts providing a response to this item. Of the survey non-responders, eleven were from mental health Trusts (46%; 20% of all mental health Trusts), seven were from acute Trusts (29%; 5% of all acute Trusts), five were from community Trusts (21%; 29% of all community Trusts) and one was from a specialist Trust (4%; 6% of all specialist Trusts).

Additionally, fifty-two Chief pharmacists also completed Survey 2 comprising the questions about the benefits of providing a helpline service.

### RE-AIM ‘adoption’

Table 1 shows the percentage of NHS Trusts in England that provide access to a patient medicines helpline, by Trust type and region. Combined, 52% of NHS Trusts provide this service (acute, 67%; specialist, 41%; mental health, 29%; and community, 18%).

**Table 1:** NHS Trusts in England providing access to a patient medicines helpline service

Type of NHS Trust/region of England	Percentage of NHS Trusts providing access to a helpline^a^
Acute Trust	67% (91/136)
Specialist Trust	41% (7/17)
Mental health Trust	29% (16/56)
Community Trust	18% (3/17)
Total NHS Trusts	52% (117/226)
East of England	72% (18/25)
South Central	69% (9/13)
South East	69% (11/16)
London	60% (21/35)
North East	60% (6/10)
Yorkshire & Humber	52% (11/21)
South West	46% (11/24)
North West	41% (16/39)
East Midlands	33% (5/15)
West Midlands	32% (9/28)

^a^Numbers in parentheses show the actual numbers of NHS Trusts that reported providing access to a helpline, out of the total number of Trusts, for the type of Trust or the region of England

Out of the 117 Trusts that provided a patient medicines helpline, 110 answered whether they operated the service directly or via another Trust. Three out of 110 Trusts reported providing the helpline service via another Trust (3%). Of the 107 Trusts which operated their own helpline, 103 Trusts operated one helpline (96%), three Trusts operated two helplines (3%) and one Trust operated three helplines (1%). Table 2 reports the percentages of where patient medicines helpline services are located within NHS Trusts, showing that helplines are predominantly located within MI centres (87%).

**Table 2:** Location of patient medicines helpline services within NHS Trusts in England

Location of the helpline service within the NHS Trust	Percentage of NHS Trusts providing their helpline from the specified location^a^
Medicines Information Centre	87% (97/112)
General clinical pharmacy services	13% (15/112)
Dispensary	4% (5/112)
Specialist clinical pharmacy services	4% (4/112)

*Note*. Nine Trusts reported that their helpline service was provided by more than one location within the NHS Trust (8%), which is why the total exceeds 100%

^a^Numbers in parentheses show the actual numbers of NHS Trusts that reported providing their helpline from the specified location, out of the total number of NHS Trusts which reported providing access to a helpline and answered this survey question

Of the 109 non-helpline Trusts, seventy-six provided comments as to why they do not offer the service. For fifty-four of the seventy-six, the reason was a lack of resources (staff time and/or funding; 71%). For sixteen of the seventy-six, the reason was not having a MI service (21%). Three Trusts answered that they do not have a helpline because they do not know what the demand would be (4%). Six per cent reported that their Trust has plans to provide a patient medicines helpline in the future, whereas 56% reported that this was a possibility, and 38% reported that their Trust did not have any plan to provide this service in the future.

Of the non-helpline Trusts, 90% reported that, if they did receive a call from a discharged patient about their medicines, they would answer the query.

### RE-AIM ‘implementation’

Tables 3, 4 and 5 shows the percentages of NHS Trusts which were found to comply with the national standards for helpline access, availability and promotion.

**Table 3:** Compliance with national standards for ‘satisfactory’ and ‘commendable’ levels of patient medicines helpline access

National standards: Helpline access		Percentage of NHS Trusts meeting each standard^a^
‘Satisfactory’ standards	Calls charged at local rate or Freephone (not a premium number).	99% (108/109)
	The phone line allows direct dialling from outside.	97% (106/109)
	An answerphone allows a message to be left outside of advertised hours.	81% (88/108)
	Contact with a pharmacy professional is always available during advertised hours.	71% (77/108)
	Total compliance with access ‘satisfactory’ standards.	54% (58/108)
‘Commendable’ standards	The helpline has a dedicated phone number.	60% (65/109)
	There is access to the service by means other than telephone, such as email, webform, personal visit^b^.	39% (42/109)
	Total compliance with access ‘commendable’ standards.	26% (28/107)
Total compliance with both ‘satisfactory’ and ‘commendable’ access standards		15% (16/107)

*Note*. Although 117 of 226 acute, mental health, specialist, and community NHS Trusts reported providing a patient medicines helpline, not all NHS Trusts answered every survey question

^a^Numbers in parentheses show the actual numbers of NHS Trusts that met the standard, out of the total number of Trusts which answered the survey question pertaining to the standard

^b^Thirty-four Trusts reported advertising their service as being accessible via one other method besides the telephone (31%), and eight Trusts reported advertising their service as being accessible via two other methods besides the telephone (7%). At thirty-four Trusts, their service was advertised as being accessible via email (31%). At eight Trusts, their service was advertised as being accessible via online web form (7%). At seven Trusts, their service was advertised as being accessible face-to-face (6%). At one Trust, their service was advertised as being accessible via social media (Twitter; 1%)

**Table 4:** Compliance with national standards for ‘satisfactory’ and ‘commendable’ levels of patient medicines helpline availability

National standards: Helpline availability		Percentage of NHS Trusts meeting each standard^a^
‘Satisfactory’ Standards	The helpline is available five days per week.	96% (103/107)
	The helpline is accessible to patients/carers for minimum of four hours per day.	86% (92/107)
	Total compliance with availability ‘satisfactory’ standards.	86% (92/107)
‘Commendable’ Standards	The helpline is available for eight hours or more per day.	57% (61/107)
	The helpline is available for extended hours (i.e., evenings, weekends^b^).	7% (7/107)
	Total compliance with availability ‘commendable’ standards.	5% (5/107)
Total compliance with both ‘satisfactory’ and ‘commendable’ availability standards		5% (5/107)

*Note*. Although 117 of 226 acute, mental health, specialist, and community NHS Trusts reported providing a patient medicines helpline, not all NHS Trusts answered every survey question

^a^Numbers in parentheses show the actual numbers of NHS Trusts that met the standard, out of the total number of Trusts which answered the survey question pertaining to the standard

^b^Three of 107 (3%) helpline services were reported as being available in the evenings; five of 107 (5%) helpline services were reported as being available at weekends (and operate seven days per week)

**Table 5:** Compliance with national standards for ‘satisfactory’ and ‘commendable’ levels of patient medicines helpline promotion

National standards: Helpline promotion		Percentage of NHS Trusts meeting each standard^a^
‘Satisfactory’ Standards	The helpline is promoted at all of the healthcare organisation’s sites.	59% (64/109)
	Promotional materials identify access times and types of enquiries patients/carers can make.	40% (44/109)
	The helpline is promoted to discharged inpatients by methods agreed with patients locally.	6% (6/100)
	Total compliance with promotion ‘satisfactory’ standards.	3% (3/100)
‘Commendable’ Standards	The helpline is also promoted to outpatients.	84% (91/108)
	Additional promotional methods are used, such as patient leaflets and the NHS Trust website^b^.	42% (46/109)
	Total compliance with promotion ‘commendable’ standards.	40% (43/108)
Total compliance with both ‘satisfactory’ and ‘commendable’ promotion standards		2% (2/99)

*Note*. Although 117 of 226 acute, mental health, specialist, and community NHS Trusts reported providing a patient medicines helpline, not all NHS Trusts answered every survey question

^a^Numbers in parentheses show the actual numbers of NHS Trusts that met the standard, out of the total number of Trusts which answered the survey question pertaining to the standard

^b^Eighty-two Trusts reported that their helpline was promoted using leaflets or business cards that are given to patients (75%). Forty-two Trusts reported that their helpline was advertised on the Trust website (38%). Forty Trusts reported that their helpline was promoted on medicines labels or on medicines bag labels (37%). Thirty-six Trusts reported that their helpline was promoted on the patient’s discharge summary (33%). Thirty Trusts reported that their helpline was promoted using posters in clinical areas (27%). Twenty-two Trusts reported that staff routinely tell patients about the helpline (20%). The median number of promotional methods used was two. The maximum number of promotional methods used by a single Trust was seven

Of the 107 NHS Trusts that answered all questions pertaining to the helpline access standards, sixteen NHS Trusts were fully compliant with all access standards (15%; 54% were compliant with all ‘satisfactory’ standards, and 26% were compliant with all ‘commendable’ standards). Of the 107 NHS Trusts that answered all questions pertaining to the helpline availability standards, five NHS Trusts were fully compliant with all availability standards (5%; 86% were compliant with all ‘satisfactory’ standards, and 5% were compliant with all ‘commendable’ standards). Of the ninety-nine NHS Trusts that answered all questions pertaining to the helpline promotion standards, two NHS Trusts were fully compliant with all promotion standards (2%; 3% were compliant with all ‘satisfactory’ standards, and 40% were compliant with all ‘commendable’ standards).

**Fig. 2:**
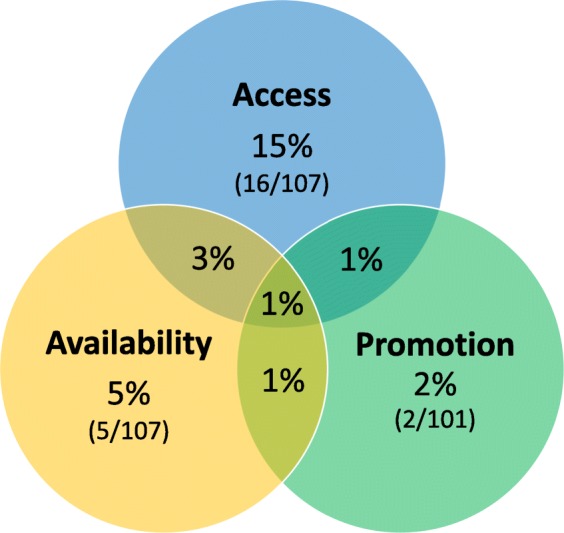
Total compliance with national standards for patient medicines helpline access, availability, and promotion. *Note*. Numbers in parentheses show the actual numbers of NHS Trusts that met the standards, out of the total number of Trusts that answered the survey questions pertaining to the standards

Out of the ninety-nine Trusts that answered all questions pertaining to the ‘satisfactory’ national standards, one NHS Trust was fully compliant with all ‘satisfactory’ standards (1%). Out of the 106 Trusts that answered all questions pertaining to the ‘commendable’ national standards, two NHS Trusts were fully compliant with all ‘commendable’ standards (2%). From a total of ninety-nine Trusts that answered all questions pertaining to both ‘satisfactory’ and ‘commendable’ national standards, one Trust was fully compliant with all standards (1%). Figure 2 shows the percentages of NHS Trusts that were found to comply with all of the national standards for helpline access, availability and promotion.

### RE-AIM ‘maintenance’

The median time that an NHS Trust had been operating a patient medicines helpline in England was six years (range 1–24 years).

Out of the 109 NHS Trusts which reported that they do not currently provide a patient medicines helpline, eighty-eight Trusts answered whether or not they provided a helpline in the past. Nine responded that they operated a helpline in the past (10%), citing main reasons for discontinuing the service as a lack of resources (lack of staff and/or funding; five of nine; 56%), and insufficient use (two of nine; 22%).

### RE-AIM ‘reach’

**Fig. 3:**
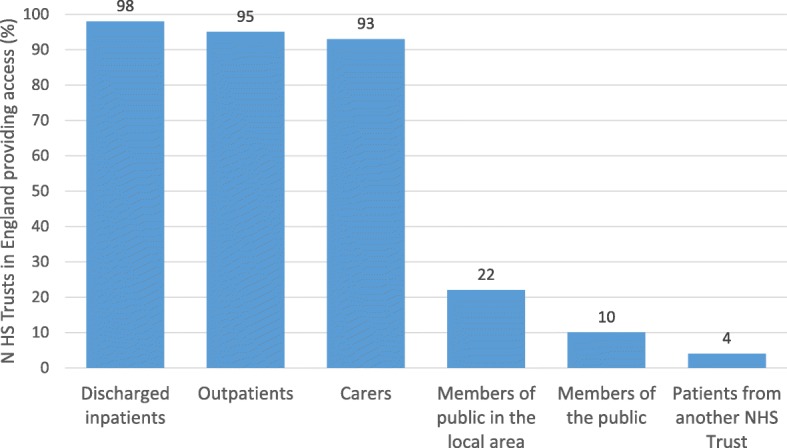
Provision of access to medicines helplines for different groups of individuals (*n* = 112)

Results showed that out of the 117 NHS Trusts that provided a patient medicines helpline, 112 Trusts answered who could access the helpline. Figure 3 shows the provision of access to medicines helplines for different groups of individuals. Medicines helplines are primarily available for discharged inpatients (98% of NHS Trusts), outpatients (95% of NHS Trusts), and patients’ carers (93% of NHS Trusts).

One hundred and seven participants reported the number of enquiries typically received to their patient medicines helpline service per week. For all Trust types combined, the median number of enquiries received per week was five (range 0–50). For acute Trusts, the median was five enquiries. For mental health Trusts, the median was three enquiries. For specialist Trusts, the median was seven enquiries. The median number of enquiries for community Trusts could not be robustly calculated due to the low number of community Trusts which operated a helpline and which answered this question.

### RE-AIM ‘effectiveness’

Table 6 provides an overview of pharmacy professionals’ perceptions regarding the major benefits of their helpline service. The top five perceived benefits were: avoiding harm to patients (88%), improving patient medication adherence (85%), providing assurance that patients can access professional help from home (83%), improving the patient experience (e.g., patient satisfaction; 80%), and supporting patient discharge (76%). Chi square tests showed that there was a significant association between professional role and benefit rating for two of the perceived benefits: avoiding harm to patients (*χ*^2^(1) = 5.65, *p* = .017), and identifying errors (*χ*^2^(1) = 9.39, *p* = .002). For both, MI pharmacy professionals were more likely to rate the benefits as being major benefits compared to Chief Pharmacists.

**Table 6:** Pharmacy professionals’ perceptions of the benefits of patient medicines helpline services

Proposed benefits of patient medicines helplines	% who see it as a major benefit		
	MI pharmacy professionals (*n* = 87)	Chief Pharmacists (*n* = 66)	Total (*n* = 156^a^)
Avoiding harm to patients (e.g., adverse effects, interactions).	93%^b^	80%^b^	88%
Improving patient medication adherence.	89%	80%	85%
Providing assurance to patients that they can access professional help from home.	84%	80%	83%
Improving the patient experience (e.g., patient satisfaction).	84%	76%	80%
Supporting patient discharge.	78%	71%	76%
Optimising medicines.	76%	73%	75%
Identifying errors.	85%^c^	64%^c^	75%
Reducing medicines-related readmissions.	67%	62%	65%
Learning from adverse patient experiences.	55%	56%	55%
Reducing visits to other healthcare services (e.g., GPs, A&E).	52%	53%	51%
Helping the organisation avoid complaints and possible litigation.	44%	42%	43%
Adhering to the NHS constitution (e.g., patients have a right to receive information).	40%	30%	37%
Improvement in Trust targets and in national surveys.	22%	26%	23%

*Note.* Although 117 of 226 acute, mental health, specialist, and community NHS Trusts reported providing a patient medicines helpline, not all NHS Trusts answered every survey question. Respondents were also provided a free-text box to record other perceived benefits. However, these suggestions were not included in the results since they were either a rewording of an item already in the list, or not also suggested by any other respondents

^a^Not all respondents provided their job title, which is why the total is greater than the number of MI pharmacy professionals and Chief Pharmacists combined

^b^A Chi square test of independence showed that there was a significant association between professional role and rating, at *p* < .05

^c^A Chi square test of independence showed that there was a significant association between professional role and rating, at *p* < .005

### Exploratory analyses

The median number of five helpline calls per week per NHS Trust was considered by our research team to be low. Exploratory analyses were conducted to explore potential ways to increase helpline use, pertaining to the areas of helpline access, availability and promotion. In order to normalise the data so that parametric tests could be conducted, the data were transformed using a log transformation. Pearson’s partial correlation coefficients were calculated to establish the relationships between the number of hours that helplines were available per week and the number of enquiries received per week, and between the number of promotional methods used and the number of enquiries received per week. The size of NHS Trusts was controlled using Hospital Episode Statistics ‘Finished Admission Episodes’ for 2015–2016 [54]. Significant positive correlations were found between the two sets of variables (*r* (95) = .31, *p* = .002 and *r* (98) = .23, *p* = .02, respectively). Additionally, an analysis of covariance was calculated to establish whether there was a statistically significant difference between the number of enquiries per week for Trusts that only provide access to their service via the telephone (mean number of enquiries per week = 7.0, SD = 8.8) versus Trusts that also provide access via at least one other method of communication (mean number of enquiries per week = 9.9, SD = 9.7). There was a significant effect of number of communication methods on the number of calls after controlling for Trust size, *F*(1, 99) = 8.89, *p* = .004, η^2^ = .073.

## Discussion

This study used the RE-AIM healthcare interventions evaluation framework to establish the provision, usage, and current practice in the operation of patient medicines helplines in NHS Trusts in England, and pharmacy professionals’ perceptions of the main benefits of their service.

Regarding the adoption of patient medicines helplines, this study shows that there is disparity of access to the service within England. Just over half of acute, mental health, specialist and community Trusts in England operate a patient medicines helpline service, although this varies according to type of Trust and region. Only 29% of mental health Trusts and 18% of community Trusts currently provide their patients with access to this service. The percentage of acute Trusts which provide a patient medicines helpline is over double that of mental health and community Trusts. This implies that the benefits of patient medicines helplines (i.e., reduced patient harm, and error correction) [26, 31, 34] are currently not experienced to the same extent for patients of mental health and community services, compared to patients of acute and specialist services. Additionally, the proportions of Trusts in the North and Midlands of England which provide the service is typically lower than the proportions of Trusts in the southern regions of England. We also found that nine Trusts reported previously operating a helpline that had been discontinued. The main reason for closure was a lack of resources/funding. Lack of resources/funding was also the main reason why 48% of Trusts did not currently provide a helpline, suggesting that this is an important barrier to providing this service. However, regarding the maintenance aspect of patient medicines helplines, our findings suggest that, once adopted, helplines are likely to become a relatively stable service for NHS Trusts. On average, NHS Trusts had been operating for six years, with the longest running for twenty-four years.

Our findings suggest that the reach of patient medicines helpline services could be improved. In the UK, up to 44% of patients who have been discharged from hospital may subsequently experience medicines-related problems [3, 4]. Given that there is an identified need for medicines information and a high number of hospital patients [54], the number of patients who use medicines helplines per week should be substantial. However, we identified that the median number of enquiries per Trust was five per week. This finding, along with similar results from previous studies, suggest that patient medicines helplines are an underused service [26–28].

Patient medicines helplines are considered to be beneficial because they have the potential to reduce the burden upon other services, including GP and A&E visits, and also to potentially reduce the number of medicines-related hospital readmissions [35]. This is topical, given that the average waiting time from booking a standard appointment to seeing a GP in England in 2016 and 2017 was estimated to be approximately two weeks [55]. Also, in the UK, the Department of Health recognises that urgent care services are struggling to cope with rising demands [40, 56]. In January 2017, and again in December 2017, the proportion of patients waiting longer than four hours in A&E reached its highest level since the collection of A&E performance data began [57]. There is also recognition that a proportion of A&E visits could be managed more appropriately elsewhere. For example, 38% of people who attend A&E receive guidance or advice only [58]. The 2014 NHS Five Year Forward View, which provides an outline for improving and modernising the NHS, emphasises that reducing the workload in A&E is a priority [40, 56]. It would therefore be beneficial to examine why patient medicines information helplines are underused, and to consider how it might be possible to encourage their use.

Our findings regarding the implementation of patient medicines helplines, whereby we compared current practice to recommended national standards for operating patient medicines helplines, may indicate why this service is underused. The access, availability, and promotion of helplines are all likely to influence their use, and we found that adherence to the national standards could be improved in all three areas. However, the greatest discrepancy between current practice and the national standards concerns the promotion of helplines. For example, promotional material containing information relating to medicine helpline access times and the types of enquiries that patients/carers can make were used in only 40% of Trusts. Not providing this information may cause frustration for callers who call outside of operating hours, and may cause confusion as to what the service provides and whether it can cater to their needs. The main reason why overall adherence to the ‘promotion’ national standards was particularly low, was because very few Trusts sought the advice of patients regarding the promotional methods to use. Including patients and carers in the development of healthcare services is increasingly recognised as being beneficial for understanding what works and why, in order to improve services [59]. Involving service users may therefore be beneficial for improving not only helpline promotion, but all aspects of this service. Our findings also suggest that increasing the number of promotional methods may increase the use of patient medicines helplines, since the number of methods was significantly correlated with number of enquiries. Additionally, helplines are typically not promoted at all hospital sites, and so this may be another potential explanation for their lack of use.

Regarding helpline access and availability, 43% of medicines helplines are available for less than eight hours a day, and at 29% of sites, a pharmacist is not always available. Therefore, service users may not be able to immediately speak to a pharmacist, and it is unknown what effect this has upon enquirers. For example, do enquirers try accessing the helpline again later or do they perhaps seek support elsewhere? We also found that approximately only 7% of Trusts that operate a helpline currently provide the service out of hours (e.g., evenings and/or weekends). For comparison, a recent survey study found that 87% of hospitals at acute and mental health Trusts in England provide an out-of-hours pharmacy advice service for healthcare professionals [60]. Our findings also show that approximately only 5% of Trusts that operate a helpline currently provide the service seven days per week. Since the number of hours per week that a helpline is open correlates with the number of enquiries received per week, another way to increase the use of patient medicines helplines may be to increase the number of hours per week that the service is available.

Our results suggest that approximately only 39% of NHS Trusts that operate a patient medicines helpline advertise the service as being accessible via at least one other method of communication besides the telephone, with the main alternative method being email. Providing access via at least one other means of communication besides the telephone was found to significantly increase the number of calls per week, albeit slightly, suggesting that this could be another way of increasing helpline use. Only one NHS Trust reported advertising their service as being accessible via social media. Service evaluation studies that have examined the types of people who call patient medicines helplines suggest that the majority are elderly [26]. In order to better engage with younger people, MI services may benefit from also providing more current methods of communication. Research carried out internationally has begun to examine alternative methods of providing MI to patients and members of the public, including online ‘Ask the Pharmacist’ services [61], and a Facebook ‘Pharmacist Hour’ [62].

Regarding the effectiveness of patient medicines helplines, we found their main perceived major benefits to be avoiding harm to patients, improving patient medication adherence, and providing assurance that patients can access professional help from home. Service evaluation studies have been conducted which provide evidence that enquiries to patient medicines helplines can result in patients avoiding harm [31], and that between 95 and 97% of enquirers subsequently report following the advice given [32, 34]. The only significant differences found between Chief Pharmacists’ and MI pharmacy professionals’ endorsements of the major benefits were for avoiding harm to patients, and identifying errors. This could be because MI pharmacy professionals have first-hand experience of interacting with helpline callers to know the types of enquiries being made and the impact they can have. Interestingly, reducing visits to other healthcare services (e.g., GPs, A&E) was considered a major benefit by only 51% of respondents. However, this could be because the number of enquiries per week per Trust was found to be relatively low, and so the reduction of visits to other services would likely be minimal (several respondents reported this as the reason for their response, in the ‘other comments’ section of the survey). Increasing the use of patient medicines helplines may shift pharmacists’ perceptions in this respect.

The list of benefits was originally developed by a small working group of proponents of patient medicines helplines [35]. This study provides stronger evidence as to the major benefits of patient medicines helplines, as perceived by a sample of 156 pharmacy professionals with expertise in patient medicines helpline provision.

### Recommendations

In order to increase the impact of patient medicines helplines, we encourage helpline providers to consider ways to increase their use. Our findings suggest that this may be achievable by improving the access, availability, and promotion of helplines. For example:

- Providing access to the service by other means in addition to the telephone, such as email, webform via the Trust website, online chat, and Skype.
- Extending the hours of availability, such as providing access to the service beyond typical 9–5 working hours (e.g., evenings and weekends).
- Increasing the number of promotional methods, and/or conducting local improvement projects to establish the types of promotional methods that patients and carers recommend, and would most likely see and remember.
- Promoting the service at all sites within the organisation, and ensuring that promotional methods identify access times and types of enquiries that can be made.

### Limitations and future research

A limitation of our study is that we were not able to obtain a full dataset, since some respondents chose not to fully complete all survey questions. Although missing data was minimal, the percentages presented in this study can only be considered to be approximately representative of the total number of NHS Trusts. Another limitation is that, in order to minimise respondent burden, we chose to only include questions that represented the sections of the national standards pertaining to helpline access, availability, and promotion. However, it would be advantageous for a future study to audit the remaining standards, since this may highlight additional ways that helpline providers may improve the delivery of their service. Subsequent research could also audit how helplines are operated in the other three UK countries, and collect additional data to explore some of the RE-AIM dimensions in greater depth. For example, a more thorough approach for understanding the reach of patient medicines helplines would be to follow up a cohort of discharged patients from Trusts that provide a medicines helpline, in order to explore those patients who subsequently require medicines information, and to compare the percentages and characteristics of helpline users with patients who choose alternative sources of support. This study design could also provide an opportunity to explore patients’ reasons for not seeking medicines information via the medicines helpline service.

Future research could also seek to establish whether and in what ways the variability in the operation of patient medicines helplines has an effect upon service users, and qualitative methods would be appropriate for exploring patients’ and carers’ experiences of using this service. Exploring the experiences that service users have regarding their medicines following their use of a patient medicines helpline could also provide further evidence as to the effectiveness of this service. Our measure of the effectiveness of helplines was limited, since it relied upon the perceptions of service providers and may be biased if participants were apprehensive about reporting any negative or poor aspects of their service. Additionally, our survey did not include a question to specifically ask pharmacy professionals to also provide their perceptions as to how patient medicines helpline services could be improved. However, our findings regarding the benefits of helplines provide a useful starting point to identify potential areas for future research. For example, studies could be designed to empirically test whether the perceived benefits of helplines are indeed benefits.

Although we have provided recommendations for increasing the use of patient medicines helplines, we acknowledge that increasing their use will likely require additional resources, and we found that a lack of staffing/funding was the main reason for NHS Trusts not providing a helpline, and for ceasing previously existing helplines. Future research could seek to establish whether a more cost-effective yet acceptable approach might be to operate a network of regional patient medicines helplines, or a national service, with collaboration from NHS Trusts for enquiries requiring local resources. However, a recent study by Badiani et al. [34] found that, out of 200 enquiries to their patient medicines helpline service, 75% required access to local knowledge. The most commonly used local source was the patients’ electronic medical records (73%), followed by contacting a healthcare professional involved in the patient’s care (34%). Badiani et al. conclude that their findings support the value of having a network of local PMHS, rather than a small number of centralised services. Further research is needed to establish the generalisability of this finding.

## Conclusion

This study demonstrates that patient medicines helplines continue to be provided by over half of NHS Trusts in England, with a similar percentage as reported by the Healthcare Commission in 2007. Also, the percentages of mental health and community Trusts that operate a helpline were found to be less than half of the percentage of acute Trusts that operate a helpline. Combined, these findings show that not all patients are able to experience the benefits that patient medicines helplines provide, due to a lack of adoption of this service. Adherence to the national standards could generally be improved, although the lowest adherence was regarding helpline promotion. Since patient medicines helplines appear to be an underused service, improving helpline access, availability and promotion may help to increase their use. However, the most cited reason for the lack of a helpline in 48% of NHS Trusts in England is lack of resources. This is also the main reason why some NHS Trusts stopped operating a helpline. Without adequate resources, it may therefore be that helpline providers do not currently have the capacity to increase the use of their service. One option could be to pool resources within regions, although this may not be possible given that many enquiry answers require local knowledge from the hospital where the patient received care. Further research is needed to explore the best way to support all patients who need help with their medicines following hospital discharge, which is cost-effective without diminishing quality.

## Additional file

Additional file 1: Survey questions. Questions and answer options for Survey 1 and Survey 2. (PDF 677 kb)

## Abbreviations

A&E: Accident and emergency
GP: General practitioner
MI: Medicines information
NHS: National Health Service
RE-AIM: Reach, Efficacy/effectiveness, adoption, implementation, and maintenance
UK: United Kingdom

## Footnotes

1 The English National Health Service (NHS) is organised in to NHS Trusts, which are organisations that provide goods and services for the purposes of health care (e.g., hospital and community services), and each Trust primarily serves a geographical area within England.
