# Supplementary material for: Operating a patient medicines helpline: a survey study exploring current practice in England using the RE-AIM evaluation framework
Source: BMC Health Serv Res. 2018 Nov 20;18:868. doi: 10.1186/s12913-018-3690-9 (PMC6245845; doi:10.1186/s12913-018-3690-9)
Supplement: Supplementary file 1 — Survey questions. Questions and answer options for Survey 1 and Survey 2. (PDF 677 kb) [file 12913_2018_3690_MOESM1_ESM.pdf]

Operating a Patient Medicines Helpline: A Survey Study Exploring Current Practice in England Using the RE-AIM Evaluation Framework

Survey 1 questions and answer options

Question	Answer type	Answer options (if applicable)
A patient medicines helpline can be defined as: "A telephone line, provided or commissioned by an NHS Trust, to enable patients and/or their carers to contact a pharmacy professional for medicines-related information and advice. It is advertised as being available for this purpose. It is specifically for medicines-related information and advice, and not for general clinical advice." Do patients and/or carers from your NHS Trust have access to a patient medicines helpline, as defined above? *(This can include a helpline which is run by your Trust, or by another provider)*	Multiple choice, single response	Yes<br>No
Questions for Trusts that provide a medicines helpline (either via their own Trust or another Trust)		
Some NHS Trusts provide patients with access to a medicines helpline which is operated from another Trust. Other NHS Trusts may operate one or more patient medicines helpline (e.g., two helplines run from different hospitals within a Trust; or a general medicines helpline for all patients, and a specialist medicines helpline for patients of a particular clinical group). How many patient medicines helplines are being operated at your NHS Trust? *(Please only include helplines which are specifically for patients to access information/advice about medicines only, rather than general clinical advice)*	Multiple choice, single response	0 (Patients and carers from my NHS Trust have access to a helpline that is provided or commissioned by another Trust).<br>1<br>2<br>3<br>4<br>5
Questions for Trusts that provide a patient medicines helpline via another organisation		
For whom at your NHS Trust is the patient medicines helpline available? *(Please read all options and tick all that apply)*	Multiple choice, multiple response	Carers of patients from my NHS Trust.<br>Discharged inpatients from my NHS Trust.<br>Outpatients from my NHS Trust.

		Any patient whose medication was prescribed and/or dispensed by my NHS Trust.<br>A specific clinical group of patients at my NHS Trust (If so, please specify in 'Other', below).<br>Other (please specify).
Who provides the patient medicines helpline? *(Please tick all that apply)*	Multiple choice, multiple response	Medicines Information Team/Service at another NHS Trust.<br>Dispensary at another NHS Trust.<br>General Clinical Pharmacy Service at another NHS Trust.<br>Specialist Clinical Pharmacy Service at another NHS Trust (If so, please specify the type of specialist service in 'Other', below).<br>Other (please specify).
From approximately what year was the patient medicines helpline service available to patients from your NHS Trust?	Free text box	
Who is the helpline *promoted* to, at your NHS Trust? *(Please tick all that apply)*	Multiple choice, multiple response	Discharged inpatients.<br>Outpatients.<br>Other (Please specify).
How is the patient medicines helpline promoted to patients at your NHS Trust? *(Please tick all that apply)*	Multiple choice, multiple response	Posters in clinical areas around the Trust.<br>On the Trust website.<br>Leaflet or business card in prescriptions.<br>On medicines labels or medicines bag labels.<br>On patients' discharge summary.<br>Staff routinely tell patients about it (e.g., discharge counselling).<br>Other (please specify).
Where is the patient medicines helpline promoted? Please tick the most relevant statement, below.	Multiple choice, single response	The helpline is promoted at all sites within the Trust.<br>The helpline is promoted at some sites within the Trust.<br>The helpline is promoted at none of the sites within the Trust.

Do the promotional materials for the patient medicines helpline advertise the following? *(Please tick all that apply)*	Multiple choice, multiple response	Days/times of the week that the helpline is available.<br>Examples of types of questions that service users can ask.<br>None of the above.
Are any of these other methods of communication advertised to patients as alternative ways to get in touch for medicines information/advice? *(Please tick all that apply)*	Multiple choice, multiple response	Email.<br>Online web form (e.g., via the Trust website).<br>Face-to-face.<br>Other (please specify).
How were the promotional methods for the patient medicines helpline decided? *(Please tick all that apply)*	Multiple choice, multiple response	Patients from my NHS Trust were consulted.<br>Recommendations from guidelines / published studies.<br>Decided by Pharmacy/MI staff at my Trust.<br>Other (please specify)
Please read the following list and tick the options that you consider to be a major benefit or a minor benefit of providing a patient medicines helpline, based upon your experience. Leave any blank if you do not consider them to be a benefit.	Multiple choice, multiple response	Avoiding harm to patients (e.g., adverse effects, interactions).<br>Identifying errors.<br>Learning from patient experiences.<br>Helping the organisation avoid complaints and possible litigation.<br>Improving patient medication adherence.<br>Supporting patient discharge.<br>Providing assurance that patients can access professional advice at home.<br>Improving the patient experience (e.g., patient satisfaction).<br>Adhering to the NHS Constitution (e.g., patients have a right to information).<br>Reducing visits to other healthcare services (e.g., GPs, A&E).<br>Reducing medicines-related readmissions.<br>Improvement in Trust targets and in national surveys (e.g., Adult Inpatient Survey).<br>Optimising medicines.

Please use the space below if you have suggestions for other benefits of providing a patient medicines helpline which aren't on the list above.	Free text box	
How do you see patient medicines helplines at NHS Trusts developing in the future? (Please consider the future at your Trust, and future changes within the NHS as a whole organisation)	Free text box	
If you have any other comments about patient medicines helplines, or about the answers you have given in this survey, please use the space below.	Free text box	
Questions for Trusts that operate at least one helpline (The majority of questions are asked about each helpline)		
Is there an agreement between your NHS Trust and any other NHS Trusts so that their patients can access the patient medicines helpline being operated at your Trust? If yes, with how many other Trusts is there such an agreement?	Multiple choice, single response	None (There isn't an agreement with any other Trusts).<br>1<br>2<br>3<br>4<br>5
For whom is your medicines helpline available? *(Please read all options and tick all that apply)*	Multiple choice, multiple response	Carers of patients from my NHS Trust.<br>Discharged inpatients from my NHS Trust.<br>Outpatients from my NHS Trust.<br>Any patient whose medication was prescribed and/or dispensed by my NHS Trust.<br>A specific clinical group of patients at my NHS Trust (If so, please specify in 'Other', below).<br>Patients from another NHS Trust (e.g., if another Trust shares your Medicines Information Team or Pharmacy Services).<br>Anyone in the local area, regardless of whether they are/were a patient or not.<br>Anyone who gets in touch, regardless of their location.<br>Other (please specify).

Who provides the patient medicines helpline? *(Please tick all that apply)*	Multiple choice, multiple response	Medicines Information Team/Service at my NHS Trust.<br>Dispensary at my NHS Trust.<br>General Clinical Pharmacy Service at my NHS Trust.<br>Specialist Clinical Pharmacy Service at my NHS Trust (If so, please specify the type of specialist service in 'Other', below).<br>Other (please specify).
Approximately what year did the patient medicines helpline first start taking calls?	Free text box	
On average, how many calls to the patient medicines helpline do you receive in a week? *(Please provide an estimate if you are unsure)*	Free text box	
Does the phone line for the patient medicines helpline allow direct dialling from outside?	Multiple choice, single response	Yes<br>No
Is the phone number for the patient medicines helpline a dedicated number, just for the helpline?	Multiple choice, single response	Yes<br>No
What is the charge for calls to the patient medicines helpline?	Multiple choice, single response	Calls are charged at a local rate.<br>Calls are charged at a premium rate.<br>Calls are free.
Who is the helpline *promoted* to, at your NHS Trust? *(Please tick all that apply)*	Multiple choice, multiple response	Discharged inpatients<br>Outpatients<br>Other (Please specify)
How is the patient medicines helpline promoted to patients at your NHS Trust? *(Please tick all that apply)*	Multiple choice, multiple response	Posters in clinical areas around the Trust.<br>On the Trust website.<br>Leaflet or business card in prescriptions.<br>On medicines labels or medicines bag labels.<br>On patients' discharge summary.

		Staff routinely tell patients about it (e.g., discharge counselling).<br>Other (please specify).
Where is the patient medicines helpline promoted? Please tick the most relevant statement, below.	Multiple choice, single response	The helpline is promoted at all sites within the Trust.<br>The helpline is promoted at some sites within the Trust.<br>The helpline is promoted at none of the sites within the Trust.
Do the promotional materials for the patient medicines helpline advertise the following? *(Please tick all that apply)*	Multiple choice, multiple response	Days/times of the week that the helpline is available.<br>Examples of types of questions that service users can ask.<br>None of the above.
Please enter the number of hours per day that the patient medicines helpline is advertised as being available, in the relevant spaces below. Please leave blank any days when the helpline is not available.	Table. Multiple choice, multiple response, with free text box.	Monday<br>Tuesday<br>Wednesday<br>Thursday<br>Friday<br>Saturday<br>Sunday
Is a pharmacy professional always available to answer calls from patients during advertised hours?	Multiple choice, single response	Yes, and there is also an answerphone service where patients can leave a message for out-of-hours calls.<br>Yes.<br>No, although there is an answerphone service where patients can leave a message.<br>No.
Are any of these other methods of communication advertised to patients as alternative ways to get in touch for medicines information/advice? *(Please tick all that apply)*	Multiple choice, multiple response	Email.<br>Online web form (e.g., via the Trust website).<br>Face-to-face.<br>Other (please specify).
How were the promotional methods for the patient medicines helpline decided? *(Please tick all that apply)*	Multiple choice, multiple response	Patients from my NHS Trust were consulted.<br>Recommendations from guidelines / published studies.

		Decided by Pharmacy/MI staff at my Trust.<br>Other (please specify)
Please read the following list and tick the options that you consider to be a major benefit or a minor benefit of providing a patient medicines helpline, based upon your experience. Leave any blank if you do not consider them to be a benefit.	Multiple choice, multiple response	Avoiding harm to patients (e.g., adverse effects, interactions).<br>Identifying errors.<br>Learning from patient experiences.<br>Helping the organisation avoid complaints and possible litigation.<br>Improving patient medication adherence.<br>Supporting patient discharge.<br>Providing assurance that patients can access professional advice at home.<br>Improving the patient experience (e.g., patient satisfaction).<br>Adhering to the NHS Constitution (e.g., patients have a right to information).<br>Reducing visits to other healthcare services (e.g., GPs, A&E).<br>Reducing medicines-related readmissions.<br>Improvement in Trust targets and in national surveys (e.g., Adult Inpatient Survey).<br>Optimising medicines.
Please use the space below if you have suggestions for other benefits of providing a patient medicines helpline which aren't on the list above.	Free text box	
How do you see patient medicines helplines at NHS Trusts developing in the future? (Please consider the future at your Trust, and future changes within the NHS as a whole organisation)	Free text box	
If you have any other comments about patient medicines helplines, or about the answers you have given in this survey, please use the space below.	Free text box	
Questions for Trusts that do not provide a patient medicines helpline (neither via their own Trust, nor outsourced)		

Has your NHS Trust provided patients with access to a medicines helpline in the past?	Multiple choice, single response	Yes<br>No
You answered that your NHS Trust provided patients to a medicines helpline in the past. Please could you provide the reason/s why the patient medicines helpline service was stopped?	Free text box	
Does your NHS Trust have any plans to provide patients with access to a medicines helpline in the future? (i.e., via your Trust, or another provider)	Multiple choice, single response	Yes<br>Possibly<br>No
Research suggests that approximately 50% of Medicines Information Centres in the UK do not operate a patient medicines helpline. We are interested to learn more about the reasons why some NHS Trusts have decided not to operate a helpline. In the space below, we would be grateful if you could please provide the reason/s why your Trust does not currently operate a patient medicines helpline.	Free text box	
If a patient was to contact the Pharmacy Services team at your NHS Trust for medicines information and advice, what would you typically do?	Multiple choice, single response	Answer their query.<br>Advise them to contact another service (e.g., their GP).<br>Other (Please specify).
How do you see patient medicines helpline services at NHS Trusts developing in the future? (Please consider future changes within the NHS as a whole organisation)	Free text box	
If you have any other comments about patient medicines helplines, or about the answers you have given in this survey, please use the space below.	Free text box	
Closing questions, for all respondents		
If you are happy to provide your job title, please enter this in the space below.	Free text box	

If you are happy to provide the name of your NHS Trust, please select it from the list below.	Drop-down list.	(List of all NHS Trusts in England, except Ambulance Trusts)
Would you potentially be interested in finding out about other research on this particular topic, carried out by the Pharmacy & Pharmacology Department at the University of Bath? This will involve receiving the occasional email about research being carried out by this specific research team only, and does not commit you to taking part. You can unsubscribe from receiving the information at any time. If so, please enter your email address below. Your email address will only be used to inform you of research studies, and will not be given to any third parties. Your contact details will not be stored alongside the other data you have provided.	Free text box	
Thank you for taking the time to complete this survey – your input is greatly appreciated. After reading this page, please click 'Finish', below, otherwise your answers will not be saved. Please tick the relevant boxes below, and if necessary provide your email address. Your email will only be used to inform you of the results and to contact you if you are randomly chosen to win the voucher, and will not be given to any third parties. Your contact details will not be stored alongside the other data you have provided.	Multiple choice, multiple response / free text box	I would like to be included in the prize draw.<br>I would like to receive the results of the study when they become available.

Operating a Patient Medicines Helpline: A Survey Study Exploring Current Practice in England Using the RE-AIM Evaluation Framework

Survey 2 questions and answer options

Question	Answer type	Answer options (if applicable)
Please read the following list and tick the options that you consider to be a major benefit or a minor benefit of providing a patient medicines helpline, based upon your experience. Leave any blank if you do not consider them to be a benefit.	Multiple choice, multiple response	Avoiding harm to patients (e.g., adverse effects, interactions).<br>Identifying errors.<br>Learning from patient experiences.<br>Helping the organisation avoid complaints and possible litigation.<br>Improving patient medication adherence.<br>Supporting patient discharge.<br>Providing assurance that patients can access professional advice at home.<br>Improving the patient experience (e.g., patient satisfaction).<br>Adhering to the NHS Constitution (e.g., patients have a right to information).<br>Reducing visits to other healthcare services (e.g., GPs, A&E).<br>Reducing medicines-related readmissions.<br>Improvement in Trust targets and in national surveys (e.g., Adult Inpatient Survey).<br>Optimising medicines.
Please use the space below if you have suggestions for other benefits of providing a patient medicines helpline which aren't on the list above.	Free text box	
How do you see patient medicines helplines at NHS Trusts developing in the future? (Please consider the future at your Trust, and future changes within the NHS as a whole organisation)	Free text box	

If you have any other comments about patient medicines helplines, or about the answers you have given in this survey, please use the space below.	Free text box	
Closing questions		
If you are happy to provide your job title, please enter this in the space below.	Free text box	
If you are happy to provide the name of your NHS Trust, please select it from the list below.	Drop-down list.	(List of all NHS Trusts in England, except Ambulance Trusts)
Would you potentially be interested in finding out about other research on this particular topic, carried out by the Pharmacy & Pharmacology Department at the University of Bath? This will involve receiving the occasional email about research being carried out by this specific research team only, and does not commit you to taking part. You can unsubscribe from receiving the information at any time. If so, please enter your email address below. Your email address will only be used to inform you of research studies, and will not be given to any third parties. Your contact details will not be stored alongside the other data you have provided.	Free text box	
Thank you for taking the time to complete this survey – your input is greatly appreciated. After reading this page, please click 'Finish', below, otherwise your answers will not be saved. Please tick the relevant boxes below, and if necessary provide your email address. Your email will only be used to inform you of the results and to contact you if you are randomly chosen to win the voucher, and will not be given to any third parties. Your contact details will not be stored alongside the other data you have provided.	Multiple choice, multiple response / free text box	I would like to be included in the prize draw.<br>I would like to receive the results of the study when they become available.
